# 

**Published:** 1882-12

**Authors:** 


### Pamphlets.

---

Some points on the administration of anæsthetics. By George H. Rohe, M.D., Baltimore.

Use of the ecraseur for curing deep-seated fistula in ano. By J. M. F. Gaston, M.D., of Campinas, Brazil. Reprint from the *American Journal of the Medical Sciences.*

Annual report of the Surgeon-General of the United States Army. By C. H. Crane, M.D., Surgeon-General.

Iodoform in surgery. By David Prince, M.D., Jacksonville, Illinois. Reprint from the *St. Louis Medical and Surgical Journal.*

